# Multimode Robust Lasing in a Dye-Doped Polymer Layer Embedded in a Wedge-Shaped Cholesteric

**DOI:** 10.3390/molecules26196089

**Published:** 2021-10-08

**Authors:** Tatevik M. Sarukhanyan, Hermine Gharagulyan, Mushegh S. Rafayelyan, Sergey S. Golik, Ashot H. Gevorgyan, Roman B. Alaverdyan

**Affiliations:** 1 Department of Physics, Yerevan State University, 1 Alex Manukyan Str., Yerevan 0025, Armenia; hermghar@gmail.com (H.G.); mrafayelyan@gmail.com (M.S.R.); ralaverdyan@ysu.am (R.B.A.); 2 Institute of Chemical Physics NAS RA, 5/2 P. Sevak Str., Yerevan 0014, Armenia; 3 Institute of Automation and Control Processes, Far Eastern Branch, Russian Academy of Sciences, 690041 Vladivostok, Russia; golik.ss@dvfu.ru; 4 School of Natural Sciences, Far Eastern Federal University, 10 Ajax Bay, Russky Island, 690922 Vladivostok, Russia; agevorgyan@ysu.am

**Keywords:** cholesteric liquid crystals, chirality, photonic band gap, polymer layer, defect modes, laser dye, lasing, geometric phase

## Abstract

Cholesteric liquid crystals (CLCs) with induced defects are one of the most prominent materials to realize compact, low-threshold and tunable coherent light sources. In this context, the investigation of optical properties of induced defect modes in such CLCs is of great interest. In particular, many studies have been devoted to the spectral control of the defect modes depending on their thickness, optical properties, distribution along the CLC, etc. In this paper, we investigate the lasing possibilities of a dye-doped polymer layer embedded in a wedge-shaped CLC. We show that multimode laser generation is possible due to the observed multiple defect modes in the PBG that enlarges the application range of the system. Furthermore, our simulations based on a Berreman 4 × 4 matrix approach for a wide range of CLC thickness show both periodic and continuous generation of defect modes along particular spectral lines inside the PBG. Such a robust spectral behaviour of induced defect modes is unique, and, to our knowledge, is not observed in similar CLC-based structures.

## 1. Introduction

Recently, special attention has been paid to the study of the optical properties of photonic structures (PS), based on liquid crystals (LC) [1]. PSs are mostly fabricated artificially allowing to create them with pre-defined properties, which, in turn, expands their functional capabilities significantly [2,3]. Cholesteric liquid crystals (CLC) are the most well-known representatives of 1D chiral PSs with local positive optical anisotropy and helical distribution of the director field [4]. Another important feature of CLC is the geometric phase which is the basis of the realization of flat mirror enabling the broadband generation of optical vortices upon reflection [5]. Furthermore, these materials self-organize their periodic structure and form a photonic bandgap (PBG) where circularly polarized light with the same handedness as that of the CLC helix cannot propagate [6]. Several studies on chiral photonic crystals have shown that it is possible to create localized optical modes within the PBG by inducing a defect into the periodic structure [7,8,9,10,11,12,13,14,15].

CLCs with induced defects are especially important for lasing possibilities, which takes place either due to the distributed feedback mechanism or defect modes [16,17,18]. Furthermore, dye-doped CLCs are widely used in tunable lasers, and have a very important role in their studies [19,20,21,22]. Although, there are many options for tuning the lasing wavelength of the dye-doped chiral nematic liquid crystal [23], the spectral tuning of laser generation in a multilayered wedge-shaped cell is less studied [21]. In [24], the authors investigated the fabrication of a wedge-cell device with a polymerizable CLC for continuous laser tuning in the full visible range by developing a continuous helical pitch gradient of the CLC in a wedge cell. Dye-doped CLC in a wedge cell is one of the best candidates to tune the lasing wavelength using the gradual change of the helical pitch along the cell. Furthermore, layered constructions give a chance to obtain multimode lasing in the broad visible spectral range due to the observed defect modes. The defect mode features depend on the position of the defect layer which influences the PBG width as well [25].

The optimization of CLC-based lasers is one of the current research directions of field experts. In [26], the authors have presented and analyzed the main strategies proposed up to now to optimize CLC lasers’ performance pointing out the main limitations regarding the cell architecture, threshold energies, and dye molecules. In [27], the micro-shell laser based on the whispering-gallery modes is considered as a vast prospective novel laser device. The authors have shown the control of the lasing modes by varying the chiral agent concentration, including PBG lasing and whispering-gallery modes, as well as the pumping energy, which can exist independently. Other interesting media for the realization of a promising laser device are polymer stabilized CLCs, and polymer dispersed CLCs for which the lasing possibilities are studied for the first time in [28]. For the design of low-threshold lasers, namely so-called edge-mode CLC lasers the study of light localization peculiarities in CLC is very important. In [29], the authors have shown that at low angles of incidence the light energy density on the long-wavelength edge mode is less than on the short-wavelength edge mode, and at large angles of incidence, there is a reverse picture.

In this context, the investigation of optical properties of CLCs with induced defects is of great interest. In particular, many above-mentioned studies have been devoted to the spectral control of the defect modes depending on their thickness, optical properties, distribution along the CLC, etc. In this paper, as a continuation to our previous work [30], we investigate the lasing possibilities of a dye-doped polymer layer (DDPL) embedded in a wedge-shaped CLC. However, in contrast to [30], the variation of the CLC thickness does not induce a pitch gradient in the cell since both boundaries between the CLC and DDPL are free of any orientation constraints. Accordingly, we show that multimode laser generation is possible due to the observed multiple defect modes in the PBG that enlarges the application range of the system. Furthermore, our simulations based on Berreman 4 × 4 matrix approach for a wide range of CLC thickness show both periodic and continuous generation of defect modes along particular spectral lines inside the PBG. Such robust spectral behaviour of induced defect modes is unique, and, to our knowledge, not observed in similar CLC-based structures.

## 2. Materials and Methods

### 2.1. Materials, Sample Preparation

**Figure 1:**
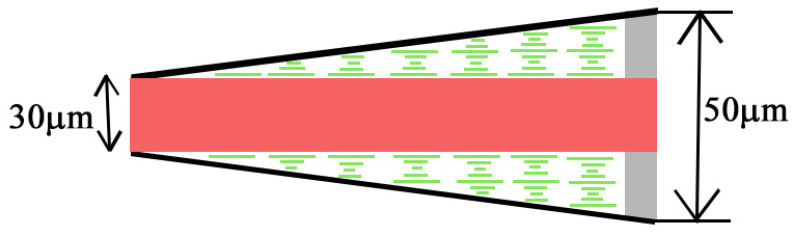
The sketch of the CLC-DDPL wedge-shaped cell.

CLC-DDPL wedge-shaped cell was prepared in the frame of this paper. Cell fabrication was started with the cleaning of the glass substrates. The next step was the coating of glass substrates with a polyimide layer as a planar aligning agent to orient the LC molecules. To obtain a uniform and thin layer of polyimide (PI), a spin coater was used firstly at 500 rpm for 5 s, and then, at 3000 rpm for the next 25 s. Afterward, the polyimide-coated substrates were rubbed in antiparallel direction using a silk coat and separated by 10 μm spacers from one side of the substrates. In the experiments, CLC mixture MDA-02-3211 with pitch *p* = 347 nm at room temperature was used. For preparing DDPL, light cure acrylic liquid polymer and rhodamine 6G (R6G) dye were used. Finally, so-called “drop-fill” method was used for the cell fabrication. The sketch of a wedge-shaped CLC cell is schematically illustrated in Figure 1.

**Figure 2:**
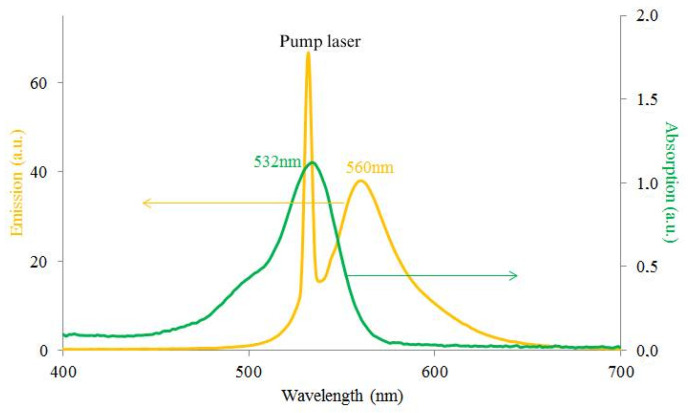
Absorption and emission spectra of DDPL.

As above mentioned, the wedge cell is comprised by a CLC material (MDA-02-3211 from Merck) and a DDPL with the 30 μm thickness embedded in the CLC. The length of the wedge cell is 30 mm and the thickness of the cell on the thin and thick sides are 30 μm and 50 μm, respectively. The average refractive index of MDA–02-3211 is $n_{a}=1.604$ (with $n_{e}=1.7013$ and $n_{o}=1.5064$ extraordinary and ordinary refractive indices, respectively) at 589.3 nm wavelength in 20 °C and it has a right-handedness [30,31]. As we know the thermal instability of liquid crystalline molecules can significantly increase the energy threshold for lasing emission, however the pitch of MDA-02-3211 has a low dependence on the temperature [32]. The refractive index of the DDPL is n=1.68. Let us mention that the isotropic layer was prepared by photopolymerization using UV light. After polymerization, the dye R6G dissolved in the polymer matrix has strong absorption in the 509–551 nm wavelength range with a maximum at 532 nm. Its emission spectrum is in the 536–579 nm wavelength range, with maximum emission near to 560 nm (Figure 2). Dye concentration in the polymer layer is $10^{-4}$mol/L to avoid possible aggregation. Our DDPL is sufficiently transparent (with low scattering) and radiation-resistant.

There are two options of using a laser dye in such structures: the molecules of laser dye are either distributed across the whole photonic structure, or they are localized in the defect layer. When laser dye is added directly to the liquid crystal several problems arise, namely due to the absorption of pumping emission the pitch of the CLC changes, and therefore the location and width of the PBG changes too. Using the second method we overcome this problem. In this way, we also bypass molecules aggregation problem, since laser dye molecules can not move in the polymer matrix.

### 2.2. Experimental Setup

**Figure 3:**
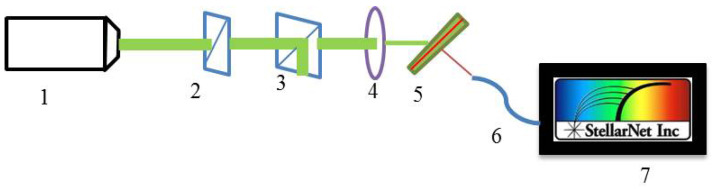
Experimental setup for investigation of laser generation in CLC-DDPL wedge-shaped system, where (1) Laser, (2) λ/2 wave plate, (3) Polarizing beam splitter, (4) Lens with 200 mm focus, (5) CLC-DDPL sample, (6) Fibre, (7) Spectrometer.

The following experimental setup was assembled (see Figure 3) to investigate the lasing possibilities and peculiarities of CLC-DDPL wedge-shaped system.

The optical pumping of the laser dye was implemented by a pulsed laser with 532 nm wavelength. The pulses duration and repetition rate are 12 ns and 12.5 Hz, respectively. The intensity of the pump laser was kept constant by 3% accuracy. The laser beam passes through the half-wave retarder and the polarizing beam splitter that controls the pumping power, and gets focused on the sample through the lens with 200 mm focal length at an angle of 45° with respect to the cell normal. Finally, the laser emission is collected by a spectrometer (StellarNet) with a resolution of 0.75 nm.

## 3. Results and Discussion

**Figure 4:**
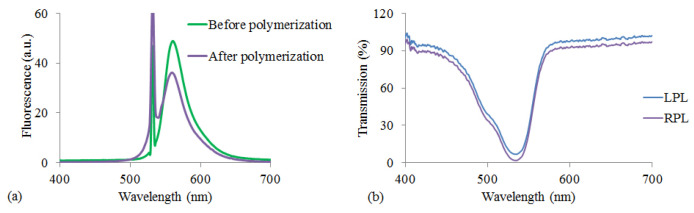
(**a**) The fluorescence spectrum of the laser dye dissolved in the polymer before and after polymerization. (**b**) Transmission spectrum from DDPL for left (LCP) and right (RCP) circularly polarized light.

We have started our experiments with studies of the fluorescence spectrum of the DDPL with the 30 μm thickness before and after polymerization (Figure 4a). As can be seen from the graph, due to the photodegradation of dye molecules the fluorescence emission decreases by photopolymerization. Furthermore, we have noticed a shift (about 2 nm) of the maximum wavelength of emission to the short-wavelength range of spectrum. However, those changes do not prevent us to provide the necessary active medium for laser generation.

We have also investigated the transmission properties of circularly polarized light in the above-mentioned layer and show that our layer is optically isotropic since the transmission coefficient for right (RCP) and left (LCP) circularly polarized light is almost the same, see Figure 4b.

**Figure 5:**
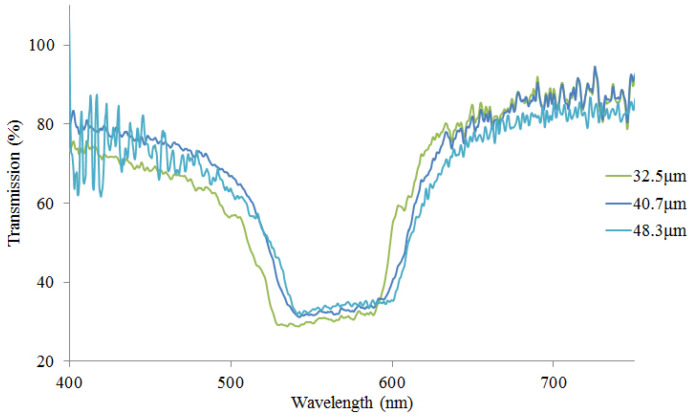
The experimentally recorded transmission spectra from CLC-DDPL wedge-shaped system corresponding to the 32.5 μm, 40.7 μm and 48.3 μm thicknesses of the cell, namely close to the edges and the intermediate part of the wedge cell (see Figure 1).

The transmission spectra of unpolarized light is recorded scanning the entire surface of the wedge-shaped cell, see Figure 5.

Scanning the wedge-shaped cell we have noticed multiple localized defect modes inside the PBG which originate due to the existence of a defect layer inside a CLC. As seen, there are multiple defect modes inside the PBG which are close to each other and averaged by a spectrometer. On the other hand, with the increase of the CLC thickness, we observe a slight change in transmission and a shift of its spectrum to the long-wavelength range because of an increase in the disordering of the CLC molecules. It is worth to mention, that with such a thick isotropic polymer layer embedded in the wedge-shaped CLC, the localized defect modes are similar to the Fabry–Perot resonator modes with diffraction mirrors. Furthermore, by changing the thickness of the CLC the orientation of the optical axis is changed in both sides of the DDPL which leads to the change of the defect modes. This is reflected also in theoretical calculations.

**Figure 6:**
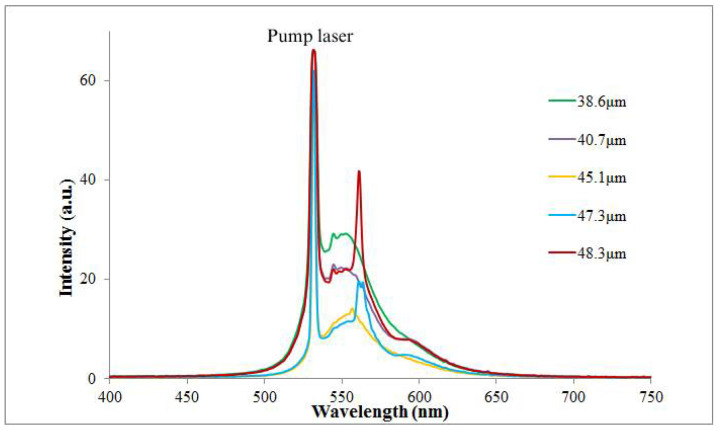
The experimentally recorded lasing generation from different thicknesses of the CLC-DDPL wedge-shaped cell. Measurements were carried out with constant 6 kW/pulse pumping energy.

Further, the lasing spectra were recorded scanning the wedge cell along its length and keeping constant the intensity of the pumping beam, see Figure 6. Since the dye-doped polymer layer is thick enough, namely 30 μm, the observed defect modes are located very close to each other, and the lasing peaks are not as narrow as expected. Note that the lasing peak at λ=532nm in the graph corresponds to the pumping laser. As seen in Figure 6, multiple lasing peaks were observed, which are originated from the defect modes of the PBG. Generated peaks from the thick side of the sample have high localization as the reflection is high enough. Further, we note that the energy distribution of the pumping beam between the lasing peaks can change with the change of the pumped region on the cell resulting in a change of localization of the peaks.

**Figure 7:**
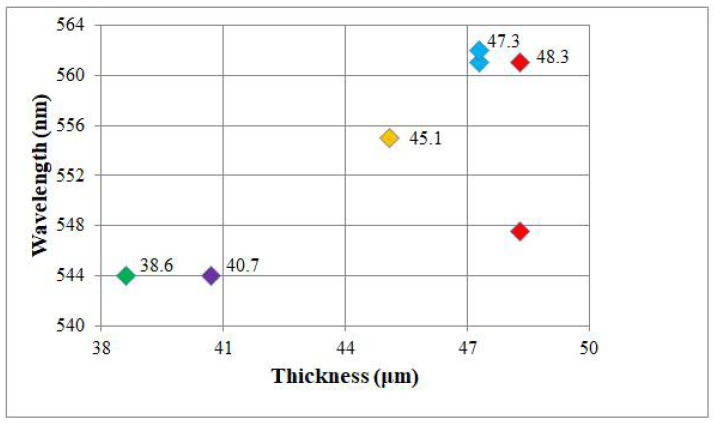
The wavelength of the generated laser peaks dependence on the thickness of the CLC-DDPL wedge-shaped cell.

To have a clear picture of the lasing peaks’ origin and their behaviour, Figure 7 shows the measured lasing peaks’ wavelengths for various thicknesses of the cell during the scanning process.

Up to two lasing peaks are observed for 48.3 μm and 47.3 μm thicknesses. Besides, some peaks are generated in the case of two different thicknesses of the sample, e.g., the laser peak with 544 nm wavelength is generated for both 38.6 μm and 40.7 μm thicknesses. However, the continuous change of the CLC thickness with the fixed thickness of DDPL eventually results in the generation of new lasing wavelengths. We note that the lasing process is repeatable and the same laser peaks appear for the same sample for the same pumping conditions. Furthermore, by using CLC oligomers it is possible to freeze the CLC-DDPL system in a glassy solid state so that the cholesteric structure and its optical properties are kept at room temperature in a perennial manner, see [33,34].

**Figure 8:**
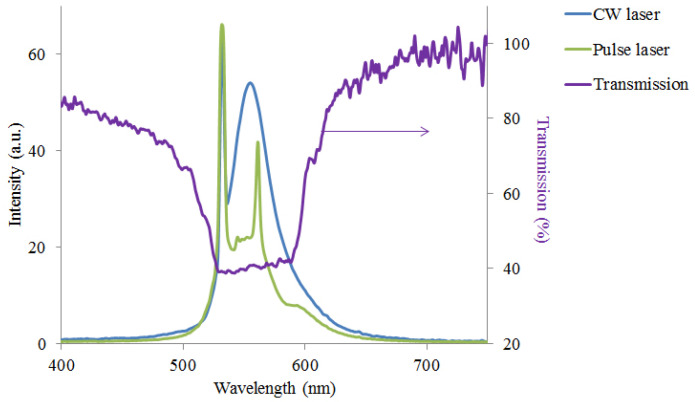
Lasing (Pulse laser), fluorescence (CW laser) and transmission spectra from the CLC-DDPL wedge-shaped cell.

To confirm that using the pulse laser we observe a lasing generation, in Figure 8 we show the measured fluorescence spectrum in the case of the continuous pump laser with 532 nm wavelength and 20 mW power. As seen, the observed spectra for continuous and pulse pumping are different, which proves that we have indeed observed a lasing generation for pump pulse laser and not a fluorescence. Furthermore, Figure 8 shows that the lasing peaks are generated on the defect modes inside the PBG. As reported in [30], the threshold pump power for lasing peaks for such a DDPL is approximately 0.8kW/pulse. However, some peaks may have a different lasing threshold since the defect modes have different light localization. We note that the handedness of the generated laser peaks is the one for which selective reflection occurs from the CLC layer. Our DDPL is isotropic and it does not have an influence on the polarization handedness of emission. So, the handedness of the laser emission from the sample remains circular which we have verified also experimentally [35].

## 4. Methods of Analysis

**Figure 9:**
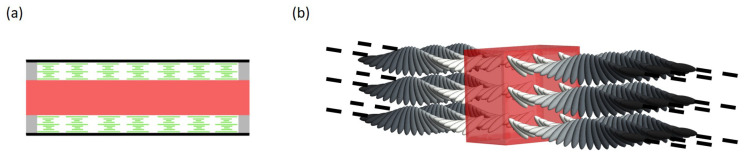
(**a**) The sketch of the CLC-IDL system considered in the theoretical simulations. (**b**) Distribution of CLC helices around the IDL showing a non-standard boundary conditions of CLC molecules.

We modelled a system consisted in an isotropic dielectric layer embedded in two equally thick CLC layers (CLC-IDL system), where the thicknesses of the CLC layers can be changed, see Figure 9a. Both boundaries of IDL are free of any orientation constraints on the CLC molecules. Thus, the optical axes orientations of CLC in both sides of a dielectric layer is defined by the thickness of CLC layers, see Figure 9b. Furthermore, the same planar boundary conditions of CLC helices on their external sides results an opposite orientation of their molecules around the isotropic layer.

**Figure 10:**
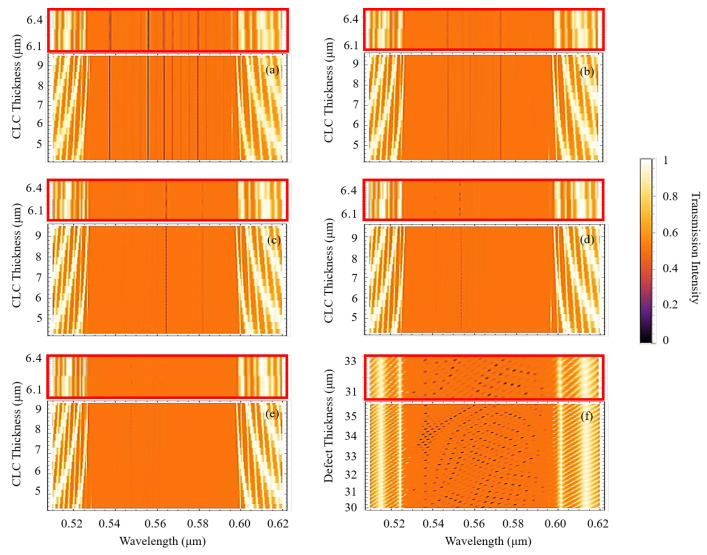
The defect modes when refractive index of defect layer is (**a**) n = 1.3, (**b**) n = 1.4, (**c**) n = 1.5, (**d**) n = 1.6 and (**e**) n = 1.68. Each panel has an inset above showing enlarged 6.1–6.4 μm region of CLC thicknesses. The CLC thickness changes from 4.3 μm to 9.5 μm. The thickness of defect layer is 30 μm. Panel (**f**) shows the transmission spectrum for defect thickness change from 30 μm to 35.8 μm, and for the CLC thickness 5.8 μm. An inset for the Panel (f) for 31 μm–33 μm is also presented.

To perform numerical calculations, we have used the Berreman 4 × 4 matrix formalism [36]. Figure 10a–e shows the transmission spectra of CLC-IDL system for the CLC layers’ thickness changing from 4.3 μm to 9.5 μm and for various refractive indices of the isotropic layer. The polarization of the incident light is taken linear in order to mimic a non-polarized incident light as in the experiment. As seen, the latter strongly affects the defect modes distribution, however in all the cases we observe either periodic or a continuous generation of defect modes along particular spectral lines inside the PBG. Such robust spectral behaviour of induced defect modes is a result of the contribution of the geometric phase induced by the multiple reflections of light from the CLC boundaries around the IDL. As known, the light reflected from such CLC structures acquires a geometric phase which is independent of wavelength and is only defined by the geometric orientations of the CLC helices, i.e., the azimuth angles of the CLC local optical axis on the reflection surface [5,31].

Accordingly, the opposite azimuth angles of CLC molecules around the isotropic layer eliminates the possibility of geometric phase accumulation during the multiple reflection of light thus ensuring a stability of defect modes over the change of the thicknesses of the CLC layers. We emphasize that the observed phenomenon is unique and related to the geometric phase nature of CLCs. As a proof, we simulated the light transmission spectrum from a similar system but instead of changing the thicknesses of the CLC layers we have changed the thickness of the IDL, see Figure 10f. As seen, the defect modes in Figure 10f shift towards long-wavelength direction over the change of the IDL thickness which is due to the dispersive nature of the isotropic layer.

Finally, to verify the experimental results, we calculated the transmission spectra of the incident plane wave in the visible region for the IDL with n=1.68 refractive index and 30 μm thickness, see Figure 10e. As seen, the defect lines in the simulation relatively well coincide with the lasing wavelengths from Figure 6 and Figure 7. We note that in the experiment the incident light on the wedge-shaped cell has a finite spot size, i.e., different rays of the light experience different thicknesses of the CLC. Furthermore, the spectrometer has a limited resolution which is 0.75 nm. Accordingly, during the experiment an averaging both over a range of thicknesses of CLC layers and over the wavelengths of the incident light is taking place which can slightly deviate the theoretical expectation from the actual experimental results.

## 5. Conclusions

In the frame of this paper, spectral peculiarities and lasing possibilities of the dye-doped polymer layer embedded in a wedge-shaped CLC cell were investigated experimentally and theoretically. Namely, multiple lasing peaks were observed, which are originating from the defect modes from the PBG. Besides, our theoretical simulations have shown that both periodic and continuous generation of defect modes along particular spectral lines inside the PBG is possible. Such robust spectral behaviour of induced defect modes is observed for the first time and it is directly related to the geometric-phase nature of CLC helices around the isotropic defect layer. One of the perspectives of our work can be the implementation of gradient-pitch CLCs in the wedge-shaped cell to widen its PBG thus widening the spectral range of multimode lasing of our system. In general, our proposed architecture opens up new application perspectives for low threshold robust lasing, multi-position triggers, filters, etc.

## Data Availability

Data sharing is not applicable to this article.

## Sample Availability

Samples of the compounds in this article are not available from the authors.
